# Recombinant Full-Length Hepatitis C Virus E1E2 Dimer Elicits Pangenotypic Neutralizing Antibodies

**DOI:** 10.3389/fimmu.2022.831285

**Published:** 2022-06-28

**Authors:** Tianli Lin, Xiaojing Chi, Xiuying Liu, Shengnan Pan, Wenfang Chen, Huarui Duan, Xinhui Zhang, Wei Yang

**Affiliations:** NHC Key Laboratory of Systems Biology of Pathogens, Institute of Pathogen Biology, Chinese Academy of Medical Sciences and Peking Union Medical College, Beijing, China

**Keywords:** hepatitis C virus, full-length envelope glycoproteins, broad-spectrum neutralizing antibody, vaccine, immunization

## Abstract

An effective prophylactic vaccine would be beneficial for controlling and eradicating hepatitis C virus (HCV) infections. However, the high diversity across HCV genotypes is a major challenge for vaccine development. Selection of the appropriate immunogen is critical to elicit broad HCV neutralizing antibodies (NAbs). To increase the antigenic coverage of heterodimer glycoproteins, we designed and produced recombinant E1E2 antigens for genotypes 1a/1b/2a/3a/6a from an IgG Fc-tagged precursor protein in FreeStyle 293-F cells. The recombinant E1 and E2 antigens were localized and associated with the endoplasmic reticulum and co-purified from membrane extracts. By examining the interactions with HCV entry co-receptors and the blockade of HCV infection, we found that these purified Fc-E1E2 proteins displayed correct folding and function. Mouse immunization results showed that each recombinant E1E2 antigen could elicit a pangenotypic antibody response to itself and other genotypes. We also found that the pentavalent formula triggered a relatively higher and more uniform NAb titer and T cell response than monovalent antigens. Taken together, our findings may provide a useful strategy for the vaccine development of HCV and other viruses with highly heterogeneous surface glycoproteins.

## Introduction

Hepatitis C virus (HCV) chronically infected approximately 71 million people worldwide as of 2015 (1), with the most common genotypes being 1, 2 and 3. HCV infection is a leading cause of liver cirrhosis and hepatocellular cancer, which seriously endangers human health and increases the disease-caused burdens in society. Although recently approved direct-acting antiviral agents (DAAs) can combat HCV infections with a cure rate of more than 95% (2), problems remain, including potential drug resistance (3, 4) and high cost of treatment (5). In addition, patients cured by DAA may be reinfected with HCV (6), and the function and metabolism of HCV-specific CD8^+^ T cells in patients are damaged (7). Most importantly, some cured individuals may still develop progressive liver failure (8–10). Therefore, there is still an urgent and unmet demand for an effective prophylactic HCV vaccine in high-risk populations (such as intravenous drug users) and in high epidemic areas.

HCV belongs to the family of Flaviviridae. Unlike the other flavivirus genus members, such as Dengue virus and Zika virus, the envelope glycoprotein of HCV is composed of E1 and E2, which usually form heterodimers on the viral surface. Despite containing the high mutation regions HVR1 and HVR2, which are prone to mutating and evading the host immune response (11), E2 mediates HCV entry and possesses many epitopes for neutralizing antibody recognition (12). In addition, neutralizing antibodies may target the E1E2 heterodimer (13, 14), suggesting that E1 might play a certain role in the immune response as an antigen. Therefore, both HCV glycoproteins are ideal antigen candidates for vaccine design. To date, the complete structure of E1E2 has not been resolved. Part of the E2 protein structure suggests that it may adopt a spherical architecture (15), which is completely different from the structure of a classical type I or type II membrane-fused protein. Therefore, exploring the immune characteristics induced by HCV E1E2 has great significance in understanding basic virology and immunology.

No licensed HCV vaccines have been approved worldwide due to multiple obstacles, including the limitations of ideal antigen design, genotype diversity, an incomplete understanding of the mechanisms of the protective immune responses, and a lack of optimal animal models. Nevertheless, arduous attempts have been made for decades to develop HCV vaccines. Many studies have shown that the clearance of acute HCV infection is largely related to the T cell immune response (16–18). Moreover, a collection of evidence has demonstrated that the presence of broadly neutralizing antibodies (bNAbs) at early stages of infection is associated with the spontaneous clearance of acute HCV infections *in vivo* (19) and *in vitro* (13, 20–23). Therefore, a prophylactic HCV vaccine that promotes both cellular and humoral immunity would be promising.

Various types of single E2- or E1E2-based vaccine candidates have been investigated, including DNA, virus-like particles (VLPs) and protein subunit vaccines. Moreover, vaccines tend to be multivalent. A quadrivalent genotype 1a/1b/2a/3a HCV VLPs vaccine (HCV core, E1 and E2) was reported to produce broader HCV-specific immune responses (24, 25), but up to 300 μg of HCV VLPs needed to be injected in pigs without an adjuvant, and up to 80 μg was immunized subcutaneously in mice. In addition, although expressed soluble E2 (sE2) might only induce low-level neutralizing antibodies (26), there was a trivalent vaccine containing sE2 from genotypes 1a, 1b and 3a that produced a pangenotype neutralizing antibody reaction in mice and rhesus monkeys (27). Each sE2 component of this trivalent vaccine elicited a unique spectrum of NAbs that acted synergistically to inhibit HCV infection, suggesting that a multivalent form of the vaccine might help to increase the coverage of the neutralizable HCV strain. However, isolated human monoclonal antibodies from patients are mainly targeted at the E2 or E1E2 heterodimer (12), which suggests that the E1E2 heterodimer may be the most ideal immunogen by mimicking authentic HCV glycoproteins. The results of a phase I clinical trial of a recombinant HCV E1E2 heterodimer vaccine showed that it elicited a multifunctional CD4^+^ T cell response and neutralizing antibody (28–31). The immunogenicity of the E1E2 heterodimer and protein purification technology make the multivalent form of the vaccine possible. Taken together, more effective vaccines need to be explored.

The aim of this study was to explore a suitable antigen vaccine for the induction of broad-spectrum neutralizing antibodies and an effective cellular immune response against HCV. Thus, we adopt either monovalent or pentavalent formulations of the full-length E1E2 heterodimer from the HCV strains H77 (GT1a), Con1 (GT1b), J6 (GT2a), S52 (GT3a) and HK6a (GT6a) to maximize the simulation of the natural conformation of envelope proteins to improve the breadth and potency of immunity, which represents the majority of HCV cases worldwide. HCV E1E2 proteins were functional and elicited HCV-specific polyclonal neutralizing antibodies in mice. Narrow superiority in the immune response between pentavalent and monovalent E1E2 suggested that conserved epitopes in the full-length E1E2 dimer may be the key elements for a promising HCV prophylactic vaccine.

## Materials and Methods

### Cell Cultures

All human hepatocyte Huh7.5.1 cells were provided by Dr. Francis Chisari (Scripps Research Institute), HEK-293T cells were obtained from ATCC, HeLa cells were obtained from ATCC, and CHO cells stably expressing human CD81 (NCBI RefSeq. NM_004356.4), or SRB1 (NCBI RefSeq. NM_001367981.1) were maintained in DMEM supplemented with penicillin, streptomycin, and 10% fetal bovine serum (Gibco, Carlsbad, CA, USA). FreeStyle 293-F cells purchased from ATCC were maintained in SMM 293-TII expression medium (Sino Biological; Beijing, China).

### Preparation of the HCVcc and HCVpp

JFH-1 genomic RNA was transcribed *in vitro*, purified with a MEGAclear Kit (Ambion), and then transfected into naïve Huh7.5.1 cells with TransMessenger Transfection Reagent (Qiagen). The supernatant that contained virus was collected and used to inoculate naïve Huh7.5.1 cells. This supernatant collection/naïve Huh7.5.1 cell inoculation process was repeated to produce a large volume HCVcc stock and was filtered (32). Retroviral pseudotypes bearing the envelope glycoproteins of HCV (HCVpp) or vesicular stomatitis virus (VSVpp) were performed as follows. Briefly, HEK293T cells were cotransfected with the envelope-deficient HIV genome pNL4-3.Luc. R^-^E^-^ and a plasmid expressing the glycoproteins of HCV or VSV, respectively. Viral supernatants were collected 60 h after transfection and filtered (32).

### Construction and Purification of the Recombinant E1E2 Antigens

We synthesized the HCV E1E2 gene by Genescript (Nanjing, China) from the codon-optimized sequences of H77 (GT1a; GenBank accession no. AF009606; amino acids 192 to 746), Con1 (GT1b; GenBank accession no. CAB46677.1; amino acids 192 to 746), J6 (GT2a; GenBank accession no. AAF01178.1; amino acids 192 to 750), S52 (GT3a; GenBank accession no. AEB71616.2; amino acids 192 to 752) and HK6a (GT6a; GenBank accession no. AEB71619.2; amino acids 192 to 752). A human IgG1 Fc fragment was inserted and fused to the N-terminus of E2 to obtain an affinity-tagged form of E1E2, and a Flag-tag was added at the N-terminus of E1 to facilitate the detection of E1 and then inserted into the pLVX lentiviral vector. Packaged lentiviruses were generated in HEK-293T cells and transduced into FreeStyle 293-F cells to obtain HEK293F cells that stably expressed Fc-E1E2 under puromycin pressure.

Recombinant Fc-E1E2 was purified from FreeStyle 293-F cell extracts using protein A Sepharose 4 Fast Flow (GE Healthcare, Piscataway, NJ) and then concentrated with a 50,000 molecular weight cutoff centrifugal filter unit (EMD Millipore, Billerica, MA). For cell extracts preparation, FreeStyle 293-F cells were collected by centrifugation at 1,000 × g for 5 min, washed twice with PBS, and ultrasonic in hypotonic solution (20 mM HEPES [pH 7.4], 1 mM EDTA, 1 mM KCl), then centrifugation was performed at 1,000 × g for 10 min at 4°C, the collected supernatant was then centrifuged at 20,000 × g for 30 min at 4°C to obtain precipitation. The pellets were then lysed with lysis buffer (100 mM NaCl, 20 mM Tris-HCl [pH 7.5], 1 mM EDTA, 0.5% Triton X-100). The lysate was further clarified by centrifugation at 20,000 × g for 25 min at 4°C and removed impurities through 0.45μM filter.

### Immunization of Mice

All animal studies were reviewed and approved by the Laboratory Animal Management and Ethics Committee at the Institute of Pathogen Biology, Chinese Academy of Medical Sciences. Female BALB/C mice were purchased from HuaFuKang Biotechnology Co., Ltd. (Beijing, China) (6 to 7 weeks old) and were cared for in accordance with institutional guidelines. Eighty female mice (20-22 g) were randomly divided into 8 groups (10 mice per group) and were injected intramuscularly at weeks 0, 2, 4 and 8 with 5 μg of the monovalent Fc-E1E2 protein of H77, Con1, J6, S52 and HK6a, respectively, the human IgG1 Fc protein (Sino Biological; Beijing, China) or the pentavalent Fc-E1E2 cocktails (a mixture of equal proportions of each monovalent Fc-E1E2) mixed with 75 μg of alum (Invitrogen, catalog no. 77161) and 7.5 μg of monophosphoryl lipid A (MPLA Vaccigrade) (Sigma Aldrich, catalog no. 699800P). Adjuvant mixed with the PBS group was set up as a control. Serum was collected at weeks 0, 9.5, 42, and 46. Sera were heat inactivated by incubation at 56°C for 1 h and kept at -80°C until use.

### Western Blotting

Monolayer cells were lysed on ice with lysis buffer (50 mM Tris-HCl [pH 7.5], 150 mM NaCl, 1% Nonidet P-40 or TxitonX-100, 2 mM EDTA) supplemented with 1% protease inhibitor cocktail (Sigma–Aldrich). Then, the lysate was cleared by centrifugation, boiled in loading buffer and loaded onto an 8 to 12% polyacrylamide gel. After electrophoresis, the separated proteins were transferred onto a nitrocellulose membrane (Bio–Rad, Hercules, CA), blocked with 10% milk for at least 1 h, and incubated with the diluted primary antibody overnight at 4°C. Secondary antibodies conjugated to IRDye 680 RD or 800CW were used for detection by the Odyssey Infrared Imaging System (LI-COR Biosciences).

### CD Spectroscopy

The final concentration of Fc-E1E2 and human IgG Fc was 10 μM in PBS. The CD spectra were acquired on a Jasco spectropolarimeter (modelJ-815; Tokyo, Japan) using a 1-nm band width with a 1-nm step resolution from 195 to 260 nm at room temperature. After correction by subtraction of a blank, data were averaged over three accumulations.

### ELISA

To analyze the capacity of Fc-E1E2 to bind to the HCV entry factor CD81, 96-well enzyme immunoassay (EIA)/radioimmunoassay (RIA) flat-bottom plates (Costar, Corning, NY, USA) were coated overnight with recombinant CD81 large extracellular loop (CD81-LEL, from Sino Biological, Beijing), and the plates were blocked with TBST buffer (25 mM Tris, 150 mM NaCl, pH 7.4, 0.05% Tween 20) containing 5% bovine serum albumin (BSA) for 1 to 3 h at 37°C. Then, the cells were incubated with different doses of Fc-E1E2 or BSA for 2 h at 37°C. The cells were incubated with horseradish peroxidase (HRP)-conjugated rabbit anti-human IgG H&L antibody (1:13000; Abcam, catalog no. ab6759) for colorimetric analysis at 450 nm in a 96-well plate reader.

### Antibody Binding Assay

To analyze the binding of AR3A antibody (20) to the Fc-E1E2, 96-well enzyme immunoassay (EIA)/radioimmunoassay (RIA) flat-bottom plates (Costar, Corning, NY, USA) were coated overnight with Fc-E1E2 (H77, Con1, J6, S52, HK6a), an equal mixture of five Fc-E1E2 proteins, BSA, hIgG Fc protein (Sino Biological, Beijing) and E1 (Sino Biological, Beijing) as control, sE2 (Sino Biological, Beijing) as positive control. The plates were blocked with TBST buffer (25 mM Tris, 150 mM NaCl, pH 7.4, 0.05% Tween 20) containing 5% bovine serum albumin (BSA) for 1 to 3 h at 37°C. Then, the cells were incubated with different doses of horseradish peroxidase (HRP)-conjugated AR3A antibody for colorimetric analysis at 450 nm in a 96-well plate reader.

### SPR Technology

The surface plasmon resonance experiments were performed at room temperature using a BiaCore T200 with CM5 sensor chips (GE Healthcare). The surfaces of the sample and reference flow cells were activated with a 1:1 mixture of 0.1 M NHS (N-hydroxysuccinimide) and 0.1 M EDC (3-(N,N-dimethylamino)propyl-N-ethylcarbodiimide) at a flow rate of 10 μL/min. The reference flow cell was left blank. All the surfaces were blocked with 1 M ethanolamine, pH 8.0. The running buffer was HBS-EP (0.01 M HEPES, pH 7.4, 150 mM NaCl, 3 mM EDTA, 0.05% surfactant P20). For the binding affinity assays, CD81-LEL was diluted in 10 mM sodium acetate buffer, pH 5.0, and immobilized on the chip at approximately 150 response units. Fc-E1E2 from H77, Con1 J6, S52 and HK6a at gradient concentrations (0, 15.625, 31.25, 62.5, 125, 250 μg/ml) flowed over the chip surface. After each cycle, the sensor surface was regenerated with 10 mM glycine-HCl pH 2.5. The data were fitted to a 1:1 interaction steady state binding model using BIA evaluation 1.0 software.

### Flow Cytometry

We performed flow cytometry analysis using CHO cells stably expressing CD81 or SR-BI. Briefly, 10 μg of Fc-E1E2 or BSA was incubated with parental CHO cells or with CHO cells expressing HCV receptors for 2 h at room temperature. After washing with PBS, the cells were incubated with PE-conjugated goat anti-human IgG Fc secondary antibody (1:100; Abcam) and then analyzed on a BD LSRII flow cytometer (BD Biosciences, San Diego, CA). The results were analyzed with FlowJo software.

### Confocal Microscopy

Immunostaining of HeLa cells was performed using antibodies against Ezrin, Erp57 (with HA tag), P4HB, E1, and E2. Briefly, Fc-E1E2 plasmids were transfected into 1 × 10^5^ HeLa cells. After 24 h, the cells were fixed with paraformaldehyde and permeated with 0.25% Triton X-100 in PBS, then incubation with 5% BSA in PBS for 1.5 hours, the cells were incubated with primary antibodies overnight at 4°C, followed by immunostaining with Alexa Fluor 594 or Alexa Fluor 488-conjugated secondary antibodies (Jackson ImmunoResearch Laboratories), or directed by Alexa Fluor 647- conjugated primary antibodies (Abcam). The nuclei were stained with 4′,6-diamidino-2-phenylindole (DAPI). Images were captured on a Leica SPW5 confocal microscope and analyzed using Leica confocal microscopy.

### HCV Infection-Blocking Assay

Huh7.5.1 cells were seeded in 96-well plates at a density of 2 × 10^4^ cells per well for 12 h. The cells were treated with serially diluted Fc-E1E2 protein or BSA protein for 1 h at 37°C, and then approximately 1 x 10^3^ focus-forming units (FFU)/ml JFH1 HCVcc was added to Huh-7.5.1 cells. After 2 h, the protein-virus mixture was changed to complete DMEM. Forty-eight hours post-infection, the cells were fixed in the original wells with paraformaldehyde and permeated with Triton X-100, followed by immunostaining with the mouse anti-HCV core antibody (1:800 dilution, Invitrogen) and IRDye secondary antibody (1:800 dilution, Li-Cor, Nebraska, USA). Images were obtained on an Odyssey Infrared Imaging System (Li-Cor, Lincoln, NE, USA).

### Antibody Titration

To measure the E1E2-specific antibody responses in serum samples by ELISA, 96-well enzyme immunoassay/radioimmunoassay flat-bottomed plates were coated overnight with 100 ng/well Fc-E1E2 (concentration was calculated by measuring absorbance at 280 nm) from H77, Con1, J6, S52, and HK6a cells and blocked in 5% BSA. Antisera from vaccinated mice were diluted in TBST containing 1% BSA and added to the plates for 2 h at 37°C followed by the addition of HRP-conjugated anti-mouse IgG secondary antibodies diluted 1:12000. Then washed 5 times in TBST buffer, TMB substrate was added to the plates to develop the color. After adding termination solution, absorbance was read at 450 nm. An absorbance with an optical density (OD) unit more than 0.1 above the prevaccination serum samples was used as the cutoff. The end point titers of the serum were defined as the reciprocal of the maximum serum dilution that had an absorbance above the cutoff.

### Neutralization Assay

For the JFH1 HCVcc neutralization assay, we performed in-cell Western blotting to assess the neutralization of the antisera. Briefly, Huh7.5.1 cells were seeded in a 96-well plate format at 2 × 10^4^ per well for 12 h, and the serum samples were heat inactivated at 56°C for 1 h. Serially diluted sera were then mixed with JFH1 HCVcc in complete DMEM to approximately 1.2 x 10^3^ FFU/ml and incubated at 37°C for 1 h. The virus-serum mixture was added to Huh-7.5.1 cells for 4 h at 37°C and replaced with complete DMEM. After 48 h, the cells were fixed and core immunostained on an Odyssey Infrared Imaging System (Li-Cor, Lincoln, NE, USA). Immunofluorescence assays were performed to quantify HCV infection by counting the number of foci.

For the HCVpp neutralization assay, HCVpp in the presence of 8 g/ml polybrene and 1 μl of 2 M HEPES (pH 7.55) was premixed with heat-inactivated diluted sera for 1 h at 37°C, and then it was added to Huh7.5 cells for 6 h and replaced with fresh culture medium. Forty-eight hours post-infection, cells were processed using the Bright-glo luciferase assay system (Promega, Madison, WI, USA). Neutralization activity was calculated by comparing the luciferase activity of immune sera to that of a prevaccination serum control at the same dilutions.

Neutralization activity was calculated using the following formula: percent neutralization= (pre - post)/pre x 100, where pre - post represents the luciferase activity done or the number of foci after incubating with either the pre- or postvaccination sera.

Serum neutralization experiments usually use two duplicate wells for each mouse. Each neutralization curve data set was normalized by the lowest dilution to define the real value for 100% neutralization. After the transformation to neutralization, the highest dilution of sera was set to 0% neutralization. Then, the neutralization curves were fitted with a nonlinear regression model to calculate the ID50 values.

### Measurement of Cellular Immune Responses by the ELISPOT Assay

Spleens were collected from mice when the animals were euthanized, and isolated splenocytes were stimulated with Fc-E1E2, medium, or human IgG Fc and then analyzed by ELISPOT. Briefly, 96-well polyvinylidene difluoride (PVDF) plates (Millipore) were precoated with anti-mouse IFN-γ capture antibody (1:200 dilution; an-18, eBiosciences) or anti-mouse IL-4 capture antibody (1:500 dilution; 11B11; eBiosciences) at 4°C overnight. Plates were blocked with 200 μL/well of Roswell Park Memorial Institute (RPMI) 1640 medium (Gibco) supplemented with 10% fetal bovine serum and 1% penicillin/streptomycin for 2 h at 37°C, and freshly isolated splenocytes were added to the plates. Fc-E1E2, human IgG Fc (control), medium (negative control), or concanavalin A (positive control) was diluted in complete RPMI 1640 medium as a stimulus at a final concentration of 10 μg/mL after incubation for 48 h. Subsequently, the plates were incubated with biotinylated anti-mouse IFN- detection antibody (R4-6A2; eBiosciences) or biotinylated anti-mouse IL-4 detection antibody (BVD6-24G2; eBiosciences) diluted in PBST for 2 h and then with alkaline phosphatase (AP)-conjugated streptavidin (Mabtech) diluted 1:1000 in PBS for 1 h. After washing, nitroblue tetrazolium (NBT)/BCIP (5-bromo-4-chloro-3-indolyl phosphate) substrate (Promega) was added to the plates and incubated for 15–30 minutes to develop the color. The cytokine-secreting cell spots were imaged and counted on a CTL Immunospot reader (Cellular Technology Ltd.)

### Statistical Analysis

Significant differences were calculated with one-way analysis of variance followed by Tukey’s multiple comparison test. Significant values are shown as follows: no significant difference *P*>0.05, *P<0.05, **P<0.01, ***P<0.001. All P value analyses were calculated by GraphPad Prism V.5.0.

## Results

### Preparation of Natively Folded Recombinant Full-Length E1E2

**Figure 1:**
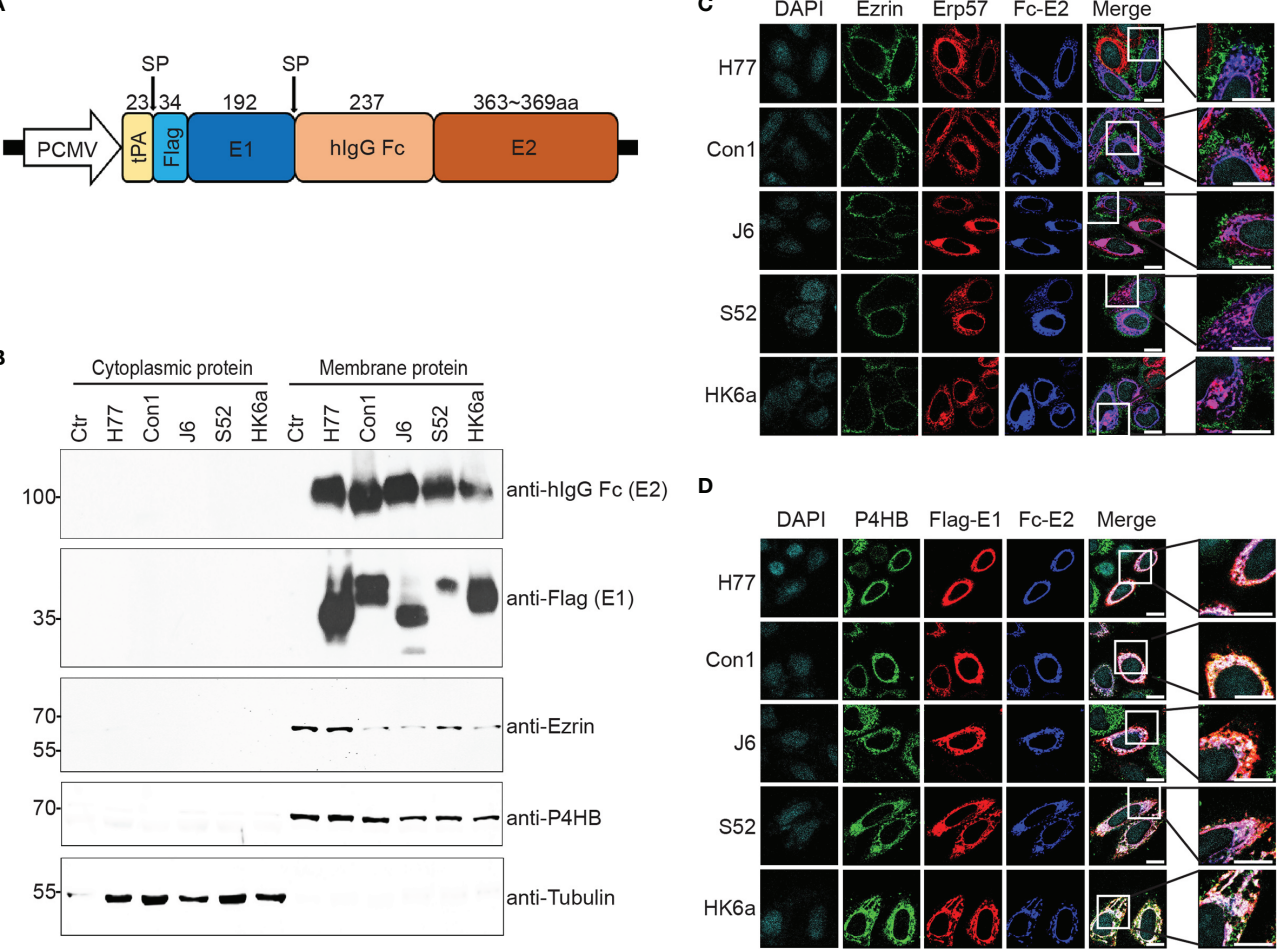
Construction, expression and purification of Fc-E1E2. **(A)** Schematic representation of the Fc-E1E2 expression plasmid. The full E1E2 was tagged with the tissue plasminogen activator (tPA) signal sequence and flag-tag at the N-terminus of E1, and the human IgG1 Fc fragment (hIgG Fc) was inserted between E1 and E2. The cleavage sites by signal peptidase (SP) and the size of the polypeptide regions (aa, amino acids) are shown at the top. **(B)** Western blotting (with anti-hIgG Fc, anti-Flag, anti-Ezrin, anti-P4HB, and anti-tubulin antibodies) of cytoplasmic and membrane extracts from FreeStyle 293-F cells. **(C, D)** The subcellular location of E2 and ER colocalization of E1 and E2 were revealed using confocal laser microscopy. The nuclei were stained with DAPI, and the ER was detected with anti-P4HB and anti-HA (Erp57 expression plasmid was tagged with HA) antibodies. The plasma membrane was detected with anti-Ezrin antibodies. Scar bar = 10μm. P4HB, Prolyl 4-hydroxylase subunit beta. Erp57, Protein disulfide isomerase family A member 3. ER, Endoplasmic reticulum. Data are shown as a representative result from at least three independent experiments.

Most subunit HCV vaccine studies are based on E2 alone. However, E1 plays an auxiliary role in stabilizing the conformation of E2. Moreover, E1 and E2 form a heterodimer that is an immunogen targeted by neutralizing antibodies (NAbs). To obtain a recombinant E1E2 heterodimer for the vaccine study, we designed and synthesized eukaryotic constructs expressing full-length E1E2 from genotypes H77 (GT1a), Con1 (GT1b), J6 (GT2a), S52 (GT3a) and HK6a (GT6a) with reference to previous studies (33). The selected genotypes represent the majority of HCV cases around the world. In contrast to previous strategies of expressing the E2 ectodomain, we expressed the full-length E1E2 molecule to facilitate the formation of heterodimer (**Figure 1A**). We characterized the expression location in subcellular compartments. As shown in **Figure 1B**, recombinant glycoproteins exclusively existed in membrane protein extracts, consistent with the membrane localization markers Ezrin and P4HB, rather than in the cytoplasmic fraction, as indicated by tubulin. We further determined the subcellular localization of recombinant E1E2 using immunofluorescent microscopy (**Figures 1C, D**). Confocal microscopy data showed that E2 protein colocalized with the endoplasmic reticulum (ER) marker Erp57, but not with plasma membrane marker Ezrin (**Figure 1C**). In addition, E1 and E2 were colocalized and merged with another ER marker P4HB (**Figure 1D**). These results provided a guidance for our subsequent recombinant protein purification.

**Figure 2:**
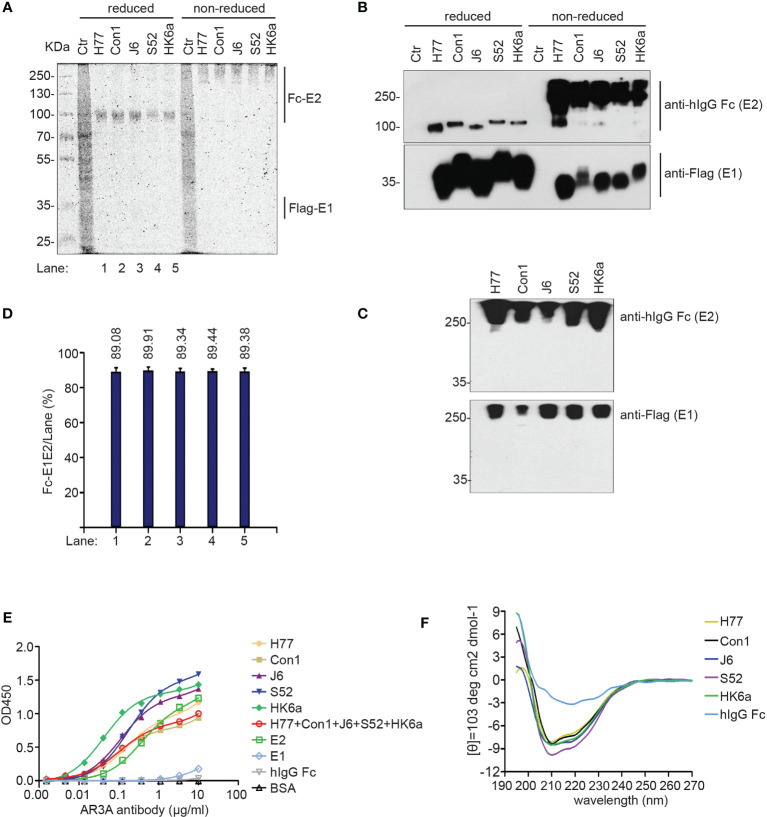
Characterization of the purified Fc-E1E2. **(A, B)** Coomassie brilliant blue staining analysis**(A)** and Western blotting analysis **(B)** of purified Fc-E1E2 from five sub-genotypes (H77, Con1, J6, S52, HK6a) under reducing and non-reducing conditions. **(C)** Native Western blotting of purified Fc-E1E2 from H77, Con1, J6, S52 and HK6a. **(D)** Data in **(D)** represent Fc-E1E2 purity from densitometry analysis to **(A)**. **(E)** AR3A antibody assay in ELISA. Precoated proteins (0.1 μg) of Fc-E1E2, BSA, E2, hIgG Fc, E1 was incubated with serially diluted AR3A antibody. Mean ± SEM of three wells were shown. **(F)** CD spectra of Fc-E1E2 from H77, Con1, J6, S52, HK6a and hIgG Fc as control in 10 μM sodium phosphate. Data are shown as a representative result from at least three independent experiments.

These E1E2 heterodimers were purified using one-step protein A Sepharose affinity purification from membrane protein extracts and were analyzed by Coomassie brilliant blue staining (**Figure 2A**) and Western blotting (**Figure 2B**) under reducing versus non-reducing loading conditions. Due to glycosylation, the molecular weight of the purified Fc-E2 protein was approximately 100 kDa under reduced conditions and above 250 kDa under non-reducing conditions, while E1 presented at approximately 35 kDa under both conditions, and the band positions of Flag-E1 and Fc-E2 were similar in native Western blotting (**Figure 2C**), indicating that E1 was copurified with the Fc-E2 protein in the form of a noncovalently linked and likely natively folded heterodimer (33). The purity of the prepared Fc-E1E2 proteins was more than 85% (**Figure 2D**), which could be used for immunization. AR3A (20), a conformation-sensitive monoclonal antibody, was used to further evaluate the E1E2 complex. Fc-E1E2 from diverse genotypes could be recognized by AR3A in a dose-dependent manner (**Figure 2E**), suggesting correctly-folded Fc-E1E2 proteins was recovered after purification. Recently, the results of both E2 core and E1 ectodomain secondary structure prediction revealed that several structured segments, specifically α-helices and β-strands conformation, are located in different regions of E1 and E2 (34). It was not clear what proportion of secondary structure the E1E2 heterodimer contained, so circular dichroism (CD) spectroscopy of E1E2 heterodimer was performed in a near physiologic solution. As shown in **Figure 2F**, unlike the human IgG Fc protein, which lacked obvious characteristic peaks, the CD spectra of five E1E2 proteins displayed signatures of double minima at 208 and 222 nm, indicating a predominantly α helical conformation in solution. The structural difference between E2 and E1E2 proteins may be partly due to the presence of the transmembrane domains of E1 and E2.

### Physical and Functional Interaction of HCV E1E2 With Cellular Receptors

**Figure 3:**
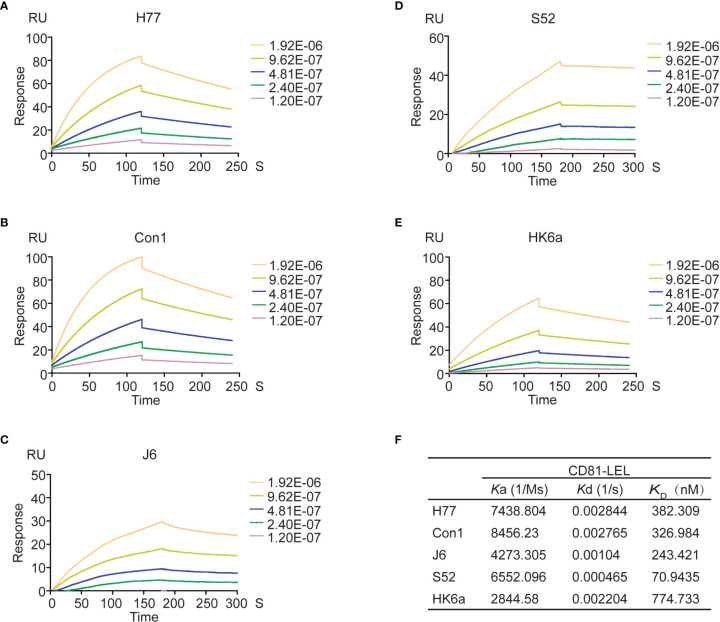
Affinity and kinetic analysis of CD81 to Fc-E1E2 proteins. **(A–E)** Sensograms for Fc-E1E2 proteins from H77, Con1, J6, S52 and HK6a binding to immobilized CD81-LEL, respectively. Fc-E1E2 proteins concentrations were 0, 15.625, 31.25, 62.5, 125, 250μg/ml. **(F)** *K*a, *K*d, *K*_D_ values of Fc-E1E2 proteins from H77, Con1, J6, S52 and HK6a binding to CD81- LEL.

A number of cell surface coreceptors, such as the tetraspanin CD81, scavenger receptor class B type I (SR-BI), claudin-1 (CLDN1) and occludin (OCLN), have been reported to be involved in essential HCV cell entry by interacting with viral glycoproteins (E1 and E2) (35). Therefore, a variety of tests were performed to characterize the functional activity of the natively folded E1E2 proteins. First, surface plasmon resonance (SPR) technology was used to measure the affinity and kinetics of E1E2 for the large extracellular loop of recombinant CD81 (CD81-LEL) (**Figure 3**). SPR results showed that the equilibrium dissociation constants (KDs) for the CD81-LEL protein against E1E2 from H77, Con1, J6, S52 and HK6a cells were 382 nM, 327 nM, 243 nM, 71 nM and 775 nM, respectively. Binding to CD81-LEL demonstrated that these glycoproteins are functional with respect to entry receptor binding critical for native HCV infection.

**Figure 4:**
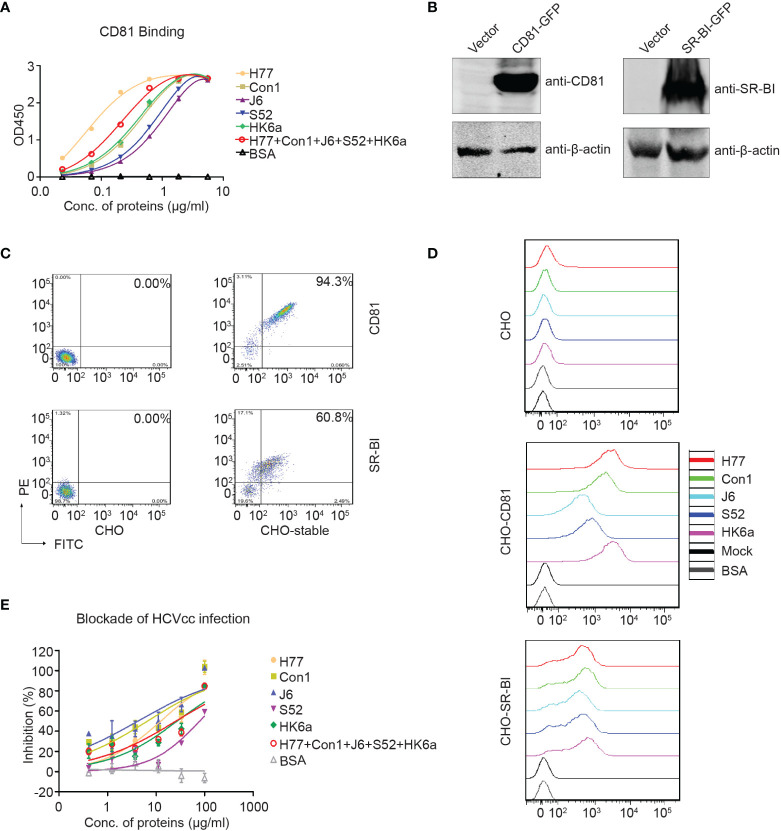
Functional characterization of the purified Fc-E1E2 proteins. **(A)** CD81 binding ELISA. Precoated CD81 (0.1 μg) was incubated with Fc-E1E2 proteins at serial concentrations. Mean ± SEM of three wells were shown. **(B, C)** CHO cells stably expressing CD81 and SR-BI, respectively. The cells were analysis by Western blotting **(B)** and flow cytometry **(C)**. **(D)** Receptor-binding assay. Fc-E1E2 protein was incubated with cells (wild-type CHO, CHO-CD81, or CHO-SR-BI), followed by PE anti-hIgG Fc antibody and analysis by flow cytometry. **(E)** Blockade of HCVcc infection by Fc-E1E2. Serially diluted Fc-E1E2 was incubated with Huh7.5.1 cells for 1 h at 37°C, and then HCVcc was added to the mixtures to allow infection for 2 h. BSA were set as control. Core immunostaining was performed at 48 h post-infection. Means and SEM of triplicates are shown, or data are shown as a representative result from three independent experiments.

Next, the association between purified E1E2 and the HCV coreceptor CD81-LEL was examined. All five E1E2 proteins reacted in a dose-dependent manner with CD81-LEL in enzyme-linked immunosorbent assay (ELISA) analysis (**Figure 4A**). H77 E1E2 presented the highest binding capacity to CD81-LEL, followed by pentavalent proteins and other genotypes. These data demonstrated that these purified E1E2 proteins have the ability to interact with CD81-LEL. The coreceptors OCLN and CLDN1 are tight junction proteins involved in the postentry stage and have indirect interactions with E1E2 (36); thus, they were not assessed for direct binding in this study. In addition to CD81, stable CHO-SR-BI cells were also created (**Figures 4B, C**). The cells were incubated with purified E1E2 and detected with flow cytometry to determine if the recombinant proteins had bound to the cells. As shown in **Figure 4D**, both CHO-CD81 and CHO-SR-BI cells showed potent binding efficiency to all five of the E1E2 proteins. To further confirm the function of the purified E1E2 proteins, we tested the inhibition of these five recombinant proteins against HCV (JFH1) infection (**Figure 4E**). The data showed that both monovalent and pentavalent proteins could block HCV infection. However, pentavalent proteins had an average inhibition efficiency of HCV infection among individual monovalent E1E2 proteins. Recombinant E1E2 proteins from H77, Con1 or J6 demonstrated the strongest inhibition, probably due to their high binding capacity to CD81-LEL. Taken together, these results confirmed that the E1E2 proteins prepared in this study are functional and can be explored as vaccine candidates for their prophylactic effects against HCV.

### Monovalent and Pentavalent E1E2s Elicited Antibody Responses in Mice

**Figure 5:**
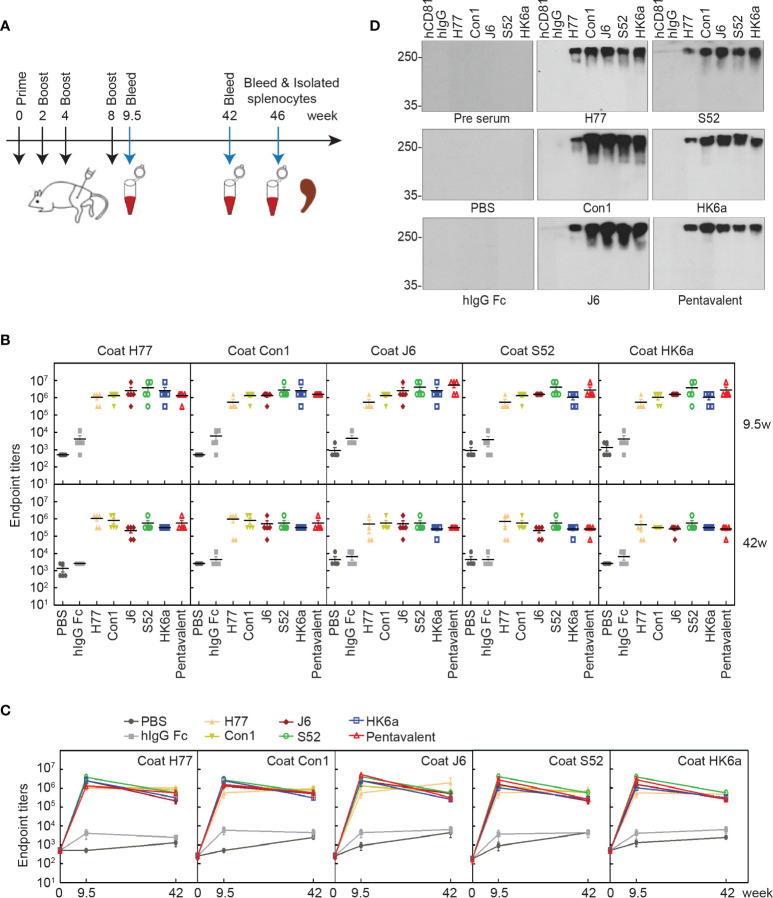
Characterization of induced antibody in mice. **(A)** Schematic representation of immunization schedule. BALB/c mice (n = 10 per group) were immunized intramuscularly at weeks 0, 2, 4, and 8, and sera were collected at weeks 9.5, 42 and 46. **(B)** Kinetics of Fc-E1E2-specific antibody titers. The specific serum titers of each group in week 9.5 and week 42, respectively. The serum titers at weeks 0, 9.5 and 42 (n = 5 per group) were measured by ELISA. The data are expressed as the mean ± SEM of the endpoint titers for each group. **(C)** Time point curve was plotted according to panel **(B)**. **(D)** Native western blotting analysis of immunized sera specificity to native Fc-E1E2. Immunized sera per group were diluted at 1:100 as the primary antibody, followed by donkey anti-mouse secondary antibody. Data are shown as a representative result from three independent experiments.

To determine the immunogenicity of these E1E2 heterodimers, BALB/c mice were immunized according to the prime-boost procedure in **Figure 5A**. As shown in **Figures 5B, C**, E1E2-specific antibody titers were approximately 10^6^ at week 9.5, and there was a slight decrease at week 42, indicating that antibody responses were elicited in mice and could last for almost the entire lifespan of mice. Interestingly, the antibody titers of each monovalent E1E2 protein showed no significant difference toward either homogenotypic or heterogenotypic antigens, suggesting that antigen design of this recombinant full-length E1E2 dimer can display conserved intergenotypic epitopes and induce a diversity of antibodies. In addition, because bNAbs mainly target conserved conformational epitopes, we performed native Western blotting to determine the antigen cross-reactivities of these immunized sera. All the immunized sera cross-reacted with native E1E2 proteins from five subgenotypes (**Figure 5D**), indicating that polyclonal antibody responses against multiple epitopes of E1E2 protein were induced in all serum immunized groups.

### Broad NAbs and Cell Immune Responses Were Elicited by Monovalent and Pentavalent E1E2s

**Figure 6:**
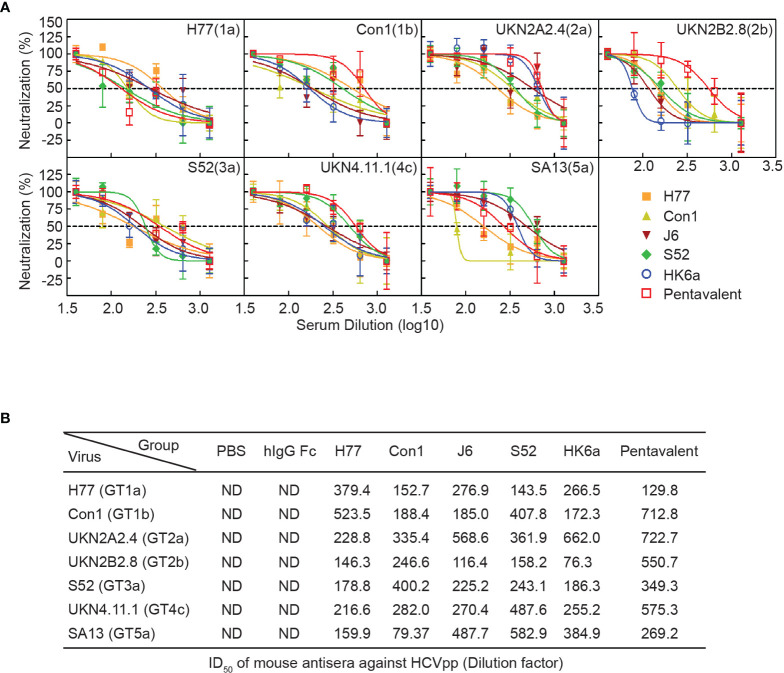
Neutralization analysis of the pentavalent and monovalent Fc-E1E2 vaccines in mice. **(A)** Neutralization curve of the week 46 vaccinated sera. Sera from each group were serially diluted two times from 40 to 1280 for the neutralization test. The neutralization curves were fitted by nonlinear regression. **(B)** ID_50_ of antisera. ID_50_ was defined as the dilution of sera able to neutralize 50% of HCVpp infectivity, and the ID_50_ values were calculated by GraphPad Prism. HCVpp, HCV pseudoparticles. ID_50_, 50% inhibitory dilution.

**Figure 7:**
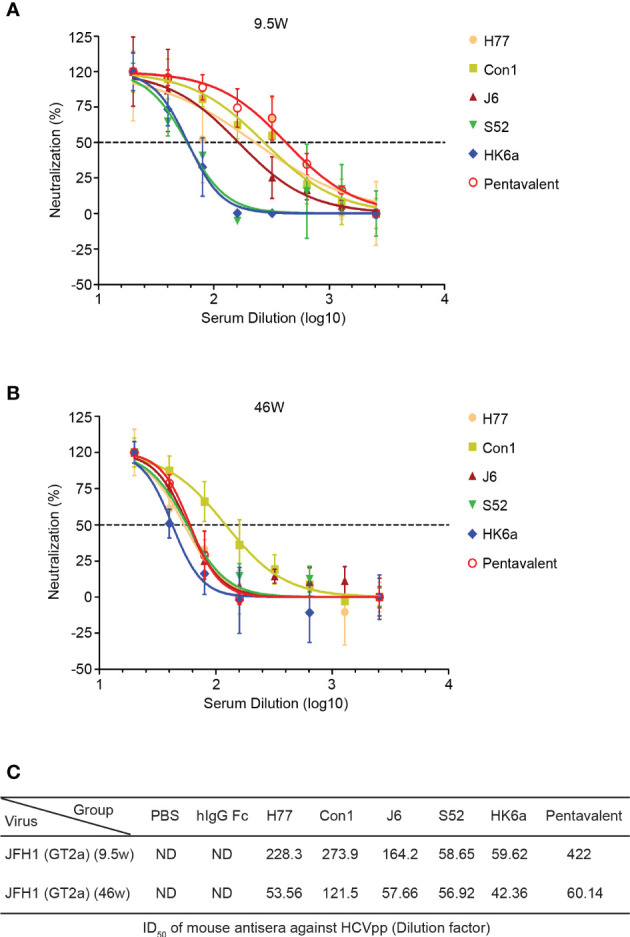
Duration of neutralization activities of the pentavalent and monovalent Fc-E1E2 vaccines in mice. **(A, B)** Neutralization curve against HCVcc of the week 9.5- and week 46-vaccinated sera, respectively. Sera from each group were serially diluted two times from 20 to 2560 for the neutralization test. The neutralization curves were fitted with a nonlinear regression model to calculate the ID_50_ values. **(C)** ID_50_ of antisera. ID_50_ was defined as the dilution of sera able to neutralize 50% of HCVcc infectivity, and the ID_50_ values were calculated by GraphPad Prism. HCVcc, cell culture-derived HCV, ID_50_, 50% inhibitory dilution.

HCV infection causes antibody responses, including neutralizing and nonneutralizing antibodies. However, the preventive effect of HCV vaccine-induced antibodies depends on the proportion of NAbs and cross-reactivity. Thus, we performed a neutralization assay using the HCV pseudoparticles (HCVpp) system to analyze the breath of immunized sera neutralization, which consisted of H77 (GT1a), Con1 (GT1b), UKN2A2.4 (GT2a), UKN2B2.8 (GT2b), S52 (GT3a), UKN4.11.1 (GT4c) and SA13 (GT5a). Due to COVID-19 restrictions, immune serum from 10 to 41 weeks could not be obtained in time, and only a small amount of 9.5-week serum was collected to detect the change in antibody titers. Therefore, pre- and week 46 serum samples were evaluated for their ability to neutralize HCVpp entry into Huh7.5.1 cells. Twofold serial dilutions of sera were examined, and the inhibitory dose to achieve the 50% inhibitory dilution (ID_50_) was represented as the reciprocal value of the dilution. As shown in **Figure 6**, the cross-neutralization ability of these immune sera against seven genotypes of pseudoviruses was measured. All immune sera showed diverse neutralization abilities for different pseudovirus genotypes. Pentavalent combination-immunized sera displayed relatively higher neutralizing efficiencies toward the above five viruses except for H77 and SA13 strains than the other immunization groups (the ID_50_ of pentavalent ranged from 349.3 to 722.7), especially for UKN2B2.8. H77-immunized sera showed sensitive neutralization reactivity against H77 and Con1 strains. UKN2A2.4 and UKN4.11.1 showed relatively sensitive to all immunized sera than other HCVpp strains. Notably, the immune sera groups did not show obvious isotype specificity against homotypic strains. These results suggested the possibility of cross neutralization activity in all E1E2-immunized sera, especially in pentavalent sera. Limited to the HCVpp panel breadth in our research, the cross-neutralization ability of immunized sera needs to be further assessed using more HCVpp subgenotypes. Idealized analyses between the diverse HCVpp and cell culture-derived HCV (HCVcc) panels should be done in parallel. However, limited by the experimental platform, only an HCVcc (JFH1 strain, genotype 2a) neutralization assay with immunized sera at week 9.5 (**Figure 7A**) and week 46 (**Figure 7B**) was performed. In the circumstance of authentic virus infection, the neutralizing activities of the pentavalent vaccine-immunized sera were much better than those of the monovalent vaccine groups, although the overall neutralization titers were relatively weak and decreased over time (**Figure 7C**). HCVpp have a tendency to be more sensitive than HCVcc to antibody neutralization (37), interestingly, the ID_50_ values of Con1-immunized sera in week 46 against H77 were close in the two panels, and Con1-immunized sera shown a slightly stronger neutralization level than pentavalent group. These findings suggest that the full-length E1E2 vaccine elicited moderate and pangenotypic polyclonal neutralizing antibodies. Among these forms, the overall performance of the pentavalent E1E2 vaccine is slightly better than that of the monovalent vaccine.

A good preventive vaccine can induce not only a strong humoral immune response but also a cellular immune response. Going forward, we measured the immune-induced antigen-specific T cellular response in splenocytes with an ELISPOT assay. At week 46 post vaccination, IL-4- and IFN-γ-secreting cells were induced in either monovalent or pentavalent vaccine-immunized groups (**Figure S2**). The IL-4 T cell response levels in the HK6a and pentavalent groups were relatively higher (**Figure S2A**), while J6, S52 and pentavalent IFN-γ T cell responses were better than those in the other immune groups (**Figure S2B**). The relatively lower proportion of IFN-γ/IL-4 in E1E2 immune groups than that in PBS immune group suggests that these E1E2-immunized groups were more inclined to inducedTh2 cell responses (**Figure S2C**). However, we did not observe consistency of genotype-specific T cell responses, suggesting that the E1E2 immunogen might induce a T cellular response against relatively conserved epitopes, and the level may be related to the existence of diverse conformations of antigens.

## Discussion

Here, we expressed Fc-E1E2 proteins, which were shown to correctly fold into heterodimers. We explored pentavalent antigens that have narrow superiority in the comprehensive performance of each test while the monovalent antigens varied, which indicated that monovalent antigens may be easier and suitable to optimize HCV vaccine design in E1E2 heterodimer form.

Immune response-induced bNAbs against HCV play an important role in the clearance of primary human HCV infections (19). Recently, dozens of bNAbs against HCV were greatly expanded, with the majority of bNAbs targeting relatively conserved HCV epitopes at the CD81 binding site of E2 (CD81bs), including discontinuous epitopes in antigenic region 3 (AR3) and a continuous epitope in antigenic site 412 (AS412) (12). In addition, the E1E2 heterodimer was also a binding site for bNAbs (12). Notably, the front layer of CD81bs is structurally flexible (38), and CD81bs, as an E2 neutralizing face, could present at least two conformations for antibody recognition (39). Proper folding of the full-length E1E2 complex might be a great immunogen for bNAb induction and binding relative to the truncated proteins (40). E1 and E2 were colocalized in the ER (**Figures 1C, D**), which is favorable for E1 and E2 glycosylation modification and permitted the formation of heterodimers in noncovalent form (**Figures 2A–E**) (41, 42). Previous evidence has shown that both of the intracellular and secreted forms of E1 and E2 can be isolated as a noncovalently linked heterodimer that represents the native intracellular forms of E1E2 (33), whereas a high-molecular-weight disulfide-linked aggregate form may represent a misfolded E1E2 (43, 44). Although previous research has shown that disulfide-linked E1E2 complexes have been detected in isolated HCV virions (45), the intracellular native form of E1E2 might replace covalently linked E1E2 on the surface of HCV virions as an ideal candidate for HCV vaccine design, as the intracellular native form of the E1E2 heterodimer facilitates purification and could elicit NAbs in healthy volunteers (30, 46, 47).

Compared with monovalent vaccines, multivalent vaccines have wider coverage and may produce synergistic effects. Similar to the polyvalent vaccine of the human papilloma virus (48), a trivalent vaccine from HCV sE2 (27) and a quadrivalent HCV VLPs vaccine (24, 25) exhibited broader immune protection. Notably, sE2 could not induce bNAbs targeting the E1E2 heterodimer because sE2 lacks the corresponding immune epitope, which might reduce the complementary neutralizing breadth and neutralizing synergy. Likewise, a relatively large amount of immunogen may not be suitable for its promotion in quadrivalent HCV VLPs vaccines. However, the pentavalent vaccine form of the HCV E1E2 heterodimer induced slightly better neutralizing antibody production (**Figures 6**, **7**) relative to the monovalent vaccine, and compared with the neutralization potency of immunized sera in week 9.5, a decrease was observed in week 46 (**Figure 7**), which may be due to the structural complexity of the E1E2 heterodimer making the superposition effect of the multivalent form-induced neutralizing antibody not prominent, although in theory, the diversity of genotypes and clusters of overlapping epitopes targeting the conserved regions of HCV envelope glycoproteins, the E1E2 vaccine, which covers multiple strains, has the possibility of eliciting NAbs targeted at multiple epitopes.

The weak signal in T cell responses and moderate neutralizing antibody response, along with the decrease in neutralization potency and titers of immunized sera over time, might be explained by the long-term duration after immunization and the short life span of mice, which means that the immune response of aging mice decreased and some of the NAbs produced after immunization were metabolized, probably illustrating that the E1E2 immunized mice had weaker NAb responses and T cell responses than with the trivalent sE2 vaccines. Factors related to immunization might also be slightly adjusted to obtain sustainable neutralization potency, such as the adjuvant types, antigen dose, interval, and so on. This shows that there is still a long way to go for the successful development of HCV vaccines.

In summary, we developed a pentavalent HCV E1E2 vaccine that is capable of eliciting slightly more potent and broader neutralizing antibodies against heterologous HCV than monovalent groups in mice. The small immunogenic superiority of the pentavalent E1E2 vaccine provides a perspective reference for further exploration of HCV vaccines inducing potent bNAbs and strong T cell responses.

## Data Availability Statement

The original contributions presented in the study are included in the article/**Supplementary Material**. Further inquiries can be directed to the corresponding author.

## Ethics Statement

The animal study was reviewed and approved by the Laboratory Animal Management and Ethics Committee at the Institute of Pathogen Biology, Chinese Academy of Medical Sciences.

## Author Contributions

WY and TL designed the experiments. TL performed most of the experiments. XC was involved in Biacore experiment. XC, XL, SP, WC, HD, and XZ discussed the data. TL and WY analyzed and interpreted the results and wrote the manuscript. WY edited the paper. All authors contributed to the article and approved the submitted version.

## Funding

This work was supported by the CAMS Innovation Fund for Medical Sciences (2021-I2M-1-038) and the National Natural Science Foundation of China (81871667).

## Conflict of Interest

The authors declare that the research was conducted in the absence of any commercial or financial relationships that could be construed as a potential conflict of interest.

## Publisher’s Note

All claims expressed in this article are solely those of the authors and do not necessarily represent those of their affiliated organizations, or those of the publisher, the editors and the reviewers. Any product that may be evaluated in this article, or claim that may be made by its manufacturer, is not guaranteed or endorsed by the publisher.
