# Supplementary material for: Recombinant Full-Length Hepatitis C Virus E1E2 Dimer Elicits Pangenotypic Neutralizing Antibodies
Source: Front Immunol. 2022 Jun 28;13:831285. doi: 10.3389/fimmu.2022.831285 (PMC9273934; doi:10.3389/fimmu.2022.831285)
Supplement: Supplementary file 1 [file DataSheet_1.docx]

**Supplementary materials**

**Figures**


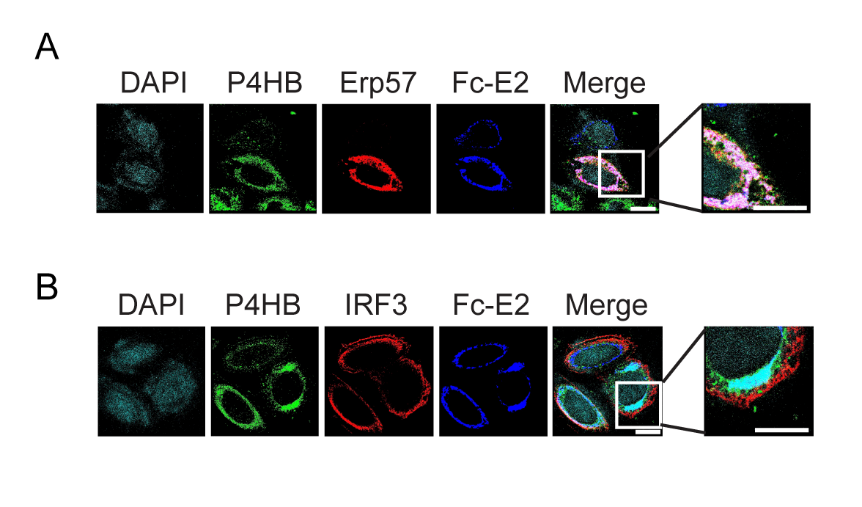


**Figure S1 Correctly localization of the plasmid encoding Erp57.** The ER location of Erp57 was revealed using confocal laser microscopy **(A)**. IRF3 was a cytoplasmic protein as a negative control **(B)**. The nuclei were stained with DAPI, and the ER was detected with anti-P4HB, Erp57 expression plasmid with HA tag was detected by anti-HA antibodies. Scar bar = 10μm. P4HB, Prolyl 4-hydroxylase subunit beta. Erp57, Protein disulfide isomerase family A member 3. ER, Endoplasmic reticulum. Data are shown as a representative result from at least three independent experiments.


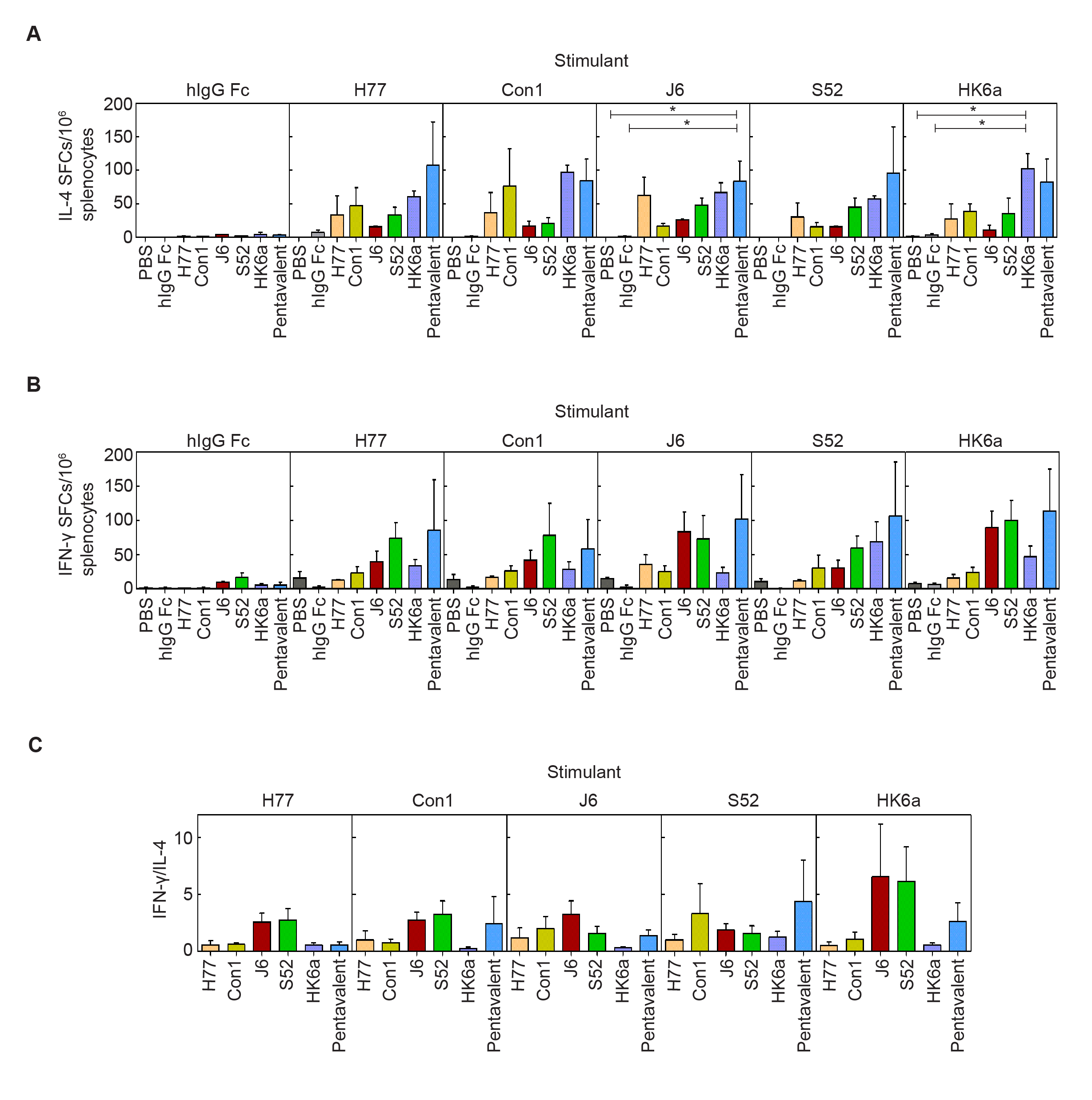


**Figure S2** **Fc-E1E2-specific T cell immune response in mice**. **(A, B)** Cellular immune responses as measured by IL-4 **(A)** and IFN-γ **(B)** ELISPOT assays. **(C)** The amount of IFN-γ relative to IL-4 was analyzed. Splenocytes were isolated from mouse spleens at week 46, followed by stimulation with purified Fc-E1E2 protein of H77, Con1, J6, S52 and HK6a. hIgG Fc as control. The results are expressed as spot-forming cells (SFCs) per 10^6^ splenocytes. The means and SEM of 3 mice within each group are shown, and the data are the average values of two replicates (n = 2). Each symbol represents an individual biological replicate. The asterisks represent significant differences (Tukey’s multiple comparison test) between groups. **P<* 0.05, ***P<* 0.01. All analyses were performed using GraphPad Prism. ELISPOT, enzyme-linked immunospot. IFN-γ, interferon gamma. IL-4, interleukin 4.

**Amino acid sequences of five E1E2 proteins**

H77

DYKDDDDKGDYKDDDDKIDYKDDDDKLEVLFQGPYQVRNSSGLYHVTNDCPNSSIVYEAADAILHTPGCVPCVREGNASRCWVAVTPTVATRDGKLPTTQLRRHIDLLVGSATLCSALYVGDLCGSVFLVGQLFTFSPRRHWTTQDCNCSIYPGHITGHRMAWDMMMNWSPTAALVVAQLLRIPQAIMDMIAGAHWGVLAGIAYFSMVGNWAKVLVVLLLFAGVDAETAPLEPKSSDKTHTCPPCPAPELLGGPSVFLFPPKPKDTLYITREPEVTCVVVDVSHEDPEVKFNWYVDGVEVHNAKTKPREEQYNSTYRVVSVLTVLHQDWLNGKEYKCKVSNKALPAPIEKTISKAKGQPREPQVYTLPPSREEMTKNQVSLTCLVKGFYPSDIAVEWESNGQPENNYKTTPPVLDSDGSFFLYSKLTVDKSRWQQGNVFSCSVMHEALKFHYTQKSLSLSPGALEVLFQGPETHVTGGNAGRTTAGLVGLLTPGAKQNIQLINTNGSWHINSTALNCNESLNTGWLAGLFYQHKFNSSGCPERLTSCRRLTDFAQGWGPISYANGSGLDERPYCWHYPPRPCGIVPAKSVCGPVYCFTPSPVVVGTTDRSGAPTYSWGANDTDVFVLNNTRPPLGNWFGCTWMNSTGFTKVCGAPPCVIGGVGNNTLLCPTDCFRKHPEATYSRCGSGPWITPRCMVDYPYRLWHYPCTINYTIFKVRMYVGGVEHRLEAACNWTRGERCDLEDRDRSELSPLLLSTTQWQVLPCSFTTLPALSTGLIHLHQNIVDVQYLYGVGSSIASWAIKWEYVVLLFLLLADARVCSCLWMMLLISQAEA

Con1

DYKDDDDKGDYKDDDDKIDYKDDDDKLEVLFQGPYEVRNVSGVYHVTNDCSNASIVYEAADMIMHTPGCVPCVRENNSSRCWVALTPTLAARNASVPTTTIRRHVDLLVGAAALCSAMYVGDLCGSVFLVAQLFTFSPRRHETVQDCNCSIYPGHVTGHRMAWDMMMNWSPTAALVVSQLLRIPQAVVDMVAGAHWGVLAGLAYYSMVGNWAKVLIVMLLFAGVDGGTAPLEPKSSDKTHTCPPCPAPELLGGPSVFLFPPKPKDTLYITREPEVTCVVVDVSHEDPEVKFNWYVDGVEVHNAKTKPREEQYNSTYRVVSVLTVLHQDWLNGKEYKCKVSNKALPAPIEKTISKAKGQPREPQVYTLPPSREEMTKNQVSLTCLVKGFYPSDIAVEWESNGQPENNYKTTPPVLDSDGSFFLYSKLTVDKSRWQQGNVFSCSVMHEALKFHYTQKSLSLSPGALEVLFQGPGTYVTGGTMAKNTLGITSLFSPGSSQKIQLVNTNGSWHINRTALNCNDSLNTGFLAALFYVHKFNSSGCPERMASCSPIDAFAQGWGPITYNESHSSDQRPYCWHYAPRPCGIVPAAQVCGPVYCFTPSPVVVGTTDRFGVPTYSWGENETDVLLLNNTRPPQGNWFGCTWMNSTGFTKTCGGPPCNIGGIGNKTLTCPTDCFRKHPEATYTKCGSGPWLTPRCLVHYPYRLWHYPCTVNFTIFKVRMYVGGVEHRLEAACNWTRGERCNLEDRDRSELSPLLLSTTEWQVLPCSFTTLPALSTGLIHLHQNVVDVQYLYGIGSAVVSFAIKWEYVLLLFLLLADARVCACLWMMLLIAQAEA

J6

DYKDDDDKGDYKDDDDKIDYKDDDDKLEVLFQGPAEVKNISTGYMVTNDCTNDSITWQLQAAVLHVPGCVPCEKVGNASQCWIPVSPNVAVQRPGALTQGLRTHIDMVVMSATLCSALYVGDLCGGVMLAAQMFIVSPQHHWFVQDCNCSIYPGTITGHRMAWDMMMNWSPTATMILAYAMRVPEVIIDIISGAHWGVMFGLAYFSMQGAWAKVVVILLLAAGVDARTAPLEPKSSDKTHTCPPCPAPELLGGPSVFLFPPKPKDTLYITREPEVTCVVVDVSHEDPEVKFNWYVDGVEVHNAKTKPREEQYNSTYRVVSVLTVLHQDWLNGKEYKCKVSNKALPAPIEKTISKAKGQPREPQVYTLPPSREEMTKNQVSLTCLVKGFYPSDIAVEWESNGQPENNYKTTPPVLDSDGSFFLYSKLTVDKSRWQQGNVFSCSVMHEALKFHYTQKSLSLSPGALEVLFQGPRTHTVGGSAAQTTGRLTSLFDMGPRQKIQLVNTNGSWHINRTALNCNDSLHTGFIASLFYTHSFNSSGCPERMSACRSIEAFRVGWGALQYEDNVTNPEDMRPYCWHYPPRQCGVVSAKTVCGPVYCFTPSPVVVGTTDRLGAPTYTWGENETDVFLLNSTRPPLGSWFGCTWMNSSGYTKTCGAPPCRTRADFNASTDLLCPTDCFRKHPDTTYLKCGSGPWLTPRCLIDYPYRLWHYPCTVNYTIFKIRMYVGGVEHRLTAACNFTRGDRCNLEDRDRSQLSPLLHSTTEWAILPCSYSDLPALSTGLLHLHQNIVDVQFMYGLSPALTKYIVRWEWVILLFLLLADARVCACLWMLILLGQAEA

S52

DYKDDDDKGDYKDDDDKIDYKDDDDKLEVLFQGPLEWRNTSGLYVLTNDCSNSSIVYEADDVILHTPGCVPCVQDDNTSTCWTPVTPTVAVRYVGATTASIRSHVDLLVGAATLCSALYVGDMCGAVFLVGQAFTFRPRRHQTVQTCNCSLYPGHVSGHRMAWDMMMNWSPAVGMVVAHILRLPQTLFDILAGAHWGILAGLAYYSMQGNWAKVAIVMIMFSGVDAETAPLEPKSSDKTHTCPPCPAPELLGGPSVFLFPPKPKDTLYITREPEVTCVVVDVSHEDPEVKFNWYVDGVEVHNAKTKPREEQYNSTYRVVSVLTVLHQDWLNGKEYKCKVSNKALPAPIEKTISKAKGQPREPQVYTLPPSREEMTKNQVSLTCLVKGFYPSDIAVEWESNGQPENNYKTTPPVLDSDGSFFLYSKLTVDKSRWQQGNVFSCSVMHEALKFHYTQKSLSLSPGALEVLFQGPETYVTGGSVAHSARGLTSLFSMGAKQKLQLVNTNGSWHINSTALNCNESINTGFIAGLFYYHKFNSTGCPQRLSSCKPIISFRQGWGPLTDANITGPSDDRPYCWHYAPRPCSVVPASSVCGPVYCFTPSPVVVGTTDIKGKPTYNWGENETDVFLLESLRPPSGRWFGCAWMNSTGFLKTCGAPPCNIYGGEGDPENETDLFCPTDCFRKHPEATYSRCGAGPWLTPRCMVDYPYRLWHYPCTVNFTLFKVRMFVGGFEHRFTAACNWTRGERCNIEDRDRSEQHPLLHSTTELAILPCSFTPMPALSTGLIHLHQNIVDVQYLYGVGSDMVGWALKWEFVILVFLLLADARVCVALWLMLMVSQAEA

HK6a

DYKDDDDKGDYKDDDDKIDYKDDDDKLEVLFQGPLTYGNSSGLYHLTNDCPNSSIVLEADAMILHLPGCLPCVRVNNNQSICWHAVSPTLAIPNASTPATGFRRHVDLLAGAAVVCSSLYIGDLCGSLFLAGQLFTFQPRRHWTVQDCNCSIYTGHVTGHRMAWDMMMNWSPTTTLVLSSILRVPEICASVISGGHWGILLAVAYFGMAGNWLKVLAVLFLFAGVEATTAPLEPKSSDKTHTCPPCPAPELLGGPSVFLFPPKPKDTLYITREPEVTCVVVDVSHEDPEVKFNWYVDGVEVHNAKTKPREEQYNSTYRVVSVLTVLHQDWLNGKEYKCKVSNKALPAPIEKTISKAKGQPREPQVYTLPPSREEMTKNQVSLTCLVKGFYPSDIAVEWESNGQPENNYKTTPPVLDSDGSFFLYSKLTVDKSRWQQGNVFSCSVMHEALKFHYTQKSLSLSPGALEVLFQGPTTTIGHQVGRTTGGLASLFSIGPRQNLQLINTTGSWHINRTALNCNDSLQTGFITSLFYAKNVNSSGCPERMAACKPLADFRQGWGQITYKVNISGPSDDRPYCWHYAPRPCDVVSARTVCGPVYCFTPSPVVVGTTDKLGIPTYNWGENETDVFMLESLRPPTGGWFGCTWMNSTGFTKTCGAPPCQIVPGDYNSSANELLCPTDCFRKHPEATYQRCGSGPWITPRCLVDYPYRLWHYPCTVNFTLHKVRMFVGGIEHRFDAACNWTRGERCDLHDRDRIEMSPLLFSTTQLAILPCSFSTMPALSTGLIHLHQNIVDVQYLYGVSSSVTSWVVKWEYIVLVFLVLADARICTCLWLMLLITNVEA
